# A rhesus macaque (*Macaca mulatta*) model of aerosol-exposure brucellosis (*Brucella suis*): pathology and diagnostic implications

**DOI:** 10.1099/jmm.0.017285-0

**Published:** 2010-06

**Authors:** Samuel L. Yingst, Louis M. Huzella, Lara Chuvala, Mark Wolcott

**Affiliations:** 1 US Army Medical Research Institute of Infectious Diseases, 1425 Porter Street, Fort Detrick, MD 21702, USA; 2 Akimeka Technologies LLC, 1425 Porter Street, Fort Detrick, MD 21702, USA

## Abstract

The US Centers for Disease Control and Prevention lists *Brucella* as a potential bioterrorism threat requiring enhanced diagnostic capacity and surveillance (http://emergency.cdc.gov/bioterrorism/). Successful treatment and management of patients after exposure to biological threat agents depends on accurate and timely diagnosis, but many biothreat agents present with similar, vague clinical signs – commonly referred to as ‘flu-like illness’. Diagnosis of brucellosis is notoriously challenging, especially early in infection, and definitive diagnosis may require invasive methods, e.g. bone marrow biopsy. We studied the pathogenesis of *Brucella suis* aerosol infection in rhesus macaques in an effort to guide the diagnostic algorithm in case of possible intentional exposure of humans. Rhesus proved to be an excellent model for human brucellosis; the data showed that PCR DNA amplification testing of non-invasive diagnostic samples has the potential to definitively detect a point-source outbreak immediately and for several days after exposure.

## INTRODUCTION

Naturally acquired human brucellosis remains common worldwide. However, the incidence in the United States and much of Europe is now very low, so brucellosis probably would not be suspected as the cause of a febrile illness in a US or European citizen with no travel history (Young, 1995). Additionally, definitive diagnosis of brucellosis has historically proven to be time-consuming and uncertain. Blood culture may be unrewarding, as viable bacteria are often undetectable in blood, and even when present, growth is slow, with blood cultures requiring up to 3 weeks of incubation before *Brucella* is detectable (Yagupsky *et al.*, 1997). Cultures on solid medium must be maintained for 3 or more days before growth is detected (Franco *et al.*, 2007). These factors may combine to reduce the probability of timely diagnosis of a point-source outbreak. When detected in the acute stage, brucellosis is much more treatable. Therefore, rapid, non-invasive diagnostic methods can be of great benefit to provide optimal opportunity for appropriate treatment and to facilitate rapid investigation in case of intentional exposure (Franz *et al.*, 2001).

PCR assays have been designed that are specific for the *Brucella* genus. Speciation by PCR is possible, but it is not essential for initial diagnostics, especially for outbreak detection. We chose to model *B. suis* for a variety of reasons that are discussed later. Naturally acquired brucellosis usually involves a low-exposure dose. In such cases, disease progression is very slow, and an association with a point source of exposure would not be expected. We did not attempt to model natural brucellosis, but instead endeavoured to model high-dose, aerosol exposure such as might occur in a bioterrorism incident. Clearly, in such a case, there would be a continuum of actual inhaled dose. We focused on the high end of this spectrum, i.e. those that would be expected to present with clinical signs soon enough for a group of illnesses to be considered a single incident.

## METHODS

### *Brucella* strain and culture.

*Brucella suis* 1330 was cultured on tryptose blood agar base slant tubes for 48 h. The slant tube was washed with 1 ml *Brucella* broth and the wash was added to a flask containing 200 ml *Brucella* broth and incubated for an additional 48 h.

### Animals, aerosol exposure, sampling, necropsy and tissue collection.

Twenty-four adult rhesus macaques (*Macaca mulatta*) were surgically implanted with Data Sciences International TA-D70 temperature and activity telemetry transmitters. Twelve macaques were assigned to this study and 12 were ‘historical controls’ from a previous study in which all handling, conditions and time points were identical. Experimental animals were exposed to *Brucella* organisms diluted in normal saline solution whereas historical controls were exposed to saline solution only. Control animals were necessary to serve as a baseline for haematology, blood chemistry and telemetry (body temperature and activity) as well as PCR, i.e. to ensure that the assay did not result in false-positives or -negatives when used on a given tissue type. Temperature and activity data were sampled every 15 min from 1 week before exposure until time of euthanasia. Complete blood counts (CBCs) and blood chemistries were performed 7, 14 and 30 days before exposure. Swab samples were taken from the face, conjunctiva, buccal mucosa, pharynx, nares and external auditory meatus and broncho-alveolar lavage (BAL) was performed immediately before exposure in order to obtain animal-matched negative control samples for PCR analysis. Extensive experience at the US Army Medical Research Institute of Infectious Diseases indicates that BAL does not affect susceptibility or disease progression in aerosol-exposed animals.

Experimental animals were exposed to approximately 1 μm mass median aerodynamic diameter aerosolized particles of *B. suis* in a manner that standardizes the number of c.f.u. inhaled. Animals were anaesthetized in accordance with institute policy. Respiratory minute volumes were estimated immediately prior to exposure using head-out plethysmography (Buxco Research Systems). Respiratory minute volume was assumed to be constant over the exposure period. Each animal was exposed separately in a well-characterized dynamic airflow exposure chamber (Dabisch *et al.*, 2010). Small particle aerosols were generated using 10 ml agent diluted in normal saline in a 3-jet Collison nebulizer (BGI). The generated aerosol was sampled using all-glass impingers attached to the exposure chamber. The contents of the impinger were assayed post-exposure to estimate the mean agent concentration in the chamber during the exposure. The estimated inhaled dose was calculated as the product of the chamber aerosol concentration, the respiratory minute volume and exposure duration. In order to achieve a targeted dose, the exposure duration was varied from animal to animal since both the chamber aerosol concentration and estimated respiratory minute volume are assumed to be constant throughout the exposure period. Exposure durations ranged from 5 to 15 min. The aerosol respiratory deposition fraction was assumed to be 100 %. Following aerosol exposure, the head of each monkey was wiped with a soap solution to remove deposited aerosol, and monkeys were housed individually under BSL-3 conditions.

Animal care was provided in accordance with established guidelines (Committee on Care and Use of Laboratory Animals of the Institute of Laboratory Animal Resources, National Research Council, 1996).

Three monkeys from each group were euthanized on days 1, 3, 5 and 7 post-exposure. On the day of euthanasia, CBC and blood chemistries, swab sampling (face, conjunctiva, buccal mucosa, pharynx, nares and external auditory meatus) and BAL were performed. During necropsy, urine was sampled by needle puncture of the exposed urinary bladder, specific organs and tissues were examined grossly, and representative samples were collected for histological evaluation. Tissue samples collected for histological evaluation were from the mandibular lymph node, liver (right caudal lobe), kidney (left), spleen, heart (ventricle), lung (right cranioventral lobe), hilar (tracheobronchial) lymph node, mesenteric lymph node, epididymis/ovarian tube, testis/ovary, prostate/uterus, ileocaecal junction, large intestine, bone marrow and brain (cerebrum).

The following testing was conducted in order to assess tissue distribution of *Brucella* and ability to detect the organism or its DNA in various samples in order to improve diagnostic methods.

### Bacterial culture.

For bacterial culture, tissues were ground with a manual tissue grinder and diluted to a 10 % concentration in PBS. Swabs were immersed in 1 ml PBS. EDTA anti-coagulated blood, serum, BAL wash fluid and urine were used without further manipulation. All samples were serially diluted up to 10-fold. Serial dilutions were plated on *Brucella* agar and incubated in 5 % CO_2_ at 37 °C in a humid incubator for 3 days. Samples were plated in triplicate and the three plate reads were averaged to generate the reported value. Any colony with the phenotypic characteristics of *Brucella* was counted as a *Brucella* organism.

### Histopathology.

Tissue samples were immersion-fixed in 10 % neutral-buffered formalin and prepared for histopathology. Sections were embedded in paraffin, sectioned, and cut at 5–6 μm, mounted on glass slides, and then stained with haematoxylin and eosin in preparation for examination by light microscopy.

### PCR.

Swab diluents, BAL wash fluid, 10 % suspensions of ground tissue samples, blood, serum and urine were extracted using the Qiagen DNA blood kit according to the manufacturer's instructions. PCR was performed on a Roche LightCycler 1.5 real-time PCR instrument as previously described using primers and probes for the *Brucella* *omp2A* gene (Christensen *et al.*, 2006). The forward and reverse primer and minor groove binder probe sequence for this assay are CCAggCgTACCggTTATCTC, AgACCCTTTTgAggTCTACTCCCTTA and TggTCgAAggCgCTC, respectively. The limit of detection of this assay is approximately 30 genome copies. Extracted DNA from *Brucella melitensis* strain 16M was used as a sample positive control. Samples were run in triplicate.

### Statistics.

Paired *t*-tests for CBC and chemistry laboratory test values at day of bleed were compared to baseline values for each day with stepdown Sidak adjustments for multiple comparisons. Three pre-bleed values for chemistry and CBC tests were averaged to obtain the baseline value for each subject.

All tissue bacterial load values were log_10_ transformed for analysis.

All temperature and activity data were log_10_ transformed for analysis. After transformation, variables were better fitted to the assumptions required for time series analysis. Temperatures and activity levels from the first 72 h were taken to be baseline values and were compared to temperatures or activity levels from the 72 h immediately before euthanasia. Telemetry of subjects euthanized on a given day (1, 3, 5 and 7) was analysed separately. Data for subjects euthanized on day 1 could not be made to fit the model due to insufficient number of time points immediately before euthanasia and were not analysed in this manner as a result. A time series model for temperature and activity was developed to examine differences between baseline temperatures or activity and temperatures or activity 72 h before euthanasia. The baseline series was used to identify an Auto-Regressive Integrated Moving Average (ARIMA) model. ARIMA is necessary to compare telemetry values obtained with a short lag between measurements, because subsequent values are influenced by previous values; regression or similar models cannot be used because independence of errors cannot be assumed. Because telemetry data were obtained every 15 min, differences were calculated at lags 1 and 96 in order to convert the raw non-stationary data to a stationary form that allows for comparison.

For direct assessment of fever spikes, fever was defined as a repeated measurement of a body temperature greater than or equal to 39.5 °C, in accordance with the institute animal care standard.

## RESULTS AND DISCUSSION

### Exposure dose

Actual inhaled dosages of *B. suis* averaged 5.60×10^8^ c.f.u. (standard error of the mean: 1.84×10^7^; range: 4.90×10^8^–6.48×10^8^).

### Demonstration of *Brucella* organisms or DNA in various samples

Nasal and/or pharyngeal swabs are common samples used to diagnose uncomplicated viral and bacterial aetiologies of febrile respiratory illnesses, whereas BAL may be ordered in cases with obvious pneumonia. Respiratory illness is a less common manifestation of naturally acquired brucellosis in humans; however, in light of the clear signs of bronchiolitis in the rhesus model under these conditions (see below), human cases occurring as a result of an intentional aerosol exposure may present with respiratory signs in addition to fever and a clinician might take nasal and pharyngeal swabs and, more rarely, BAL, without considering brucellosis to be a differential diagnosis. *Brucella* organisms were detected by routine culture (see Methods) and *Brucella* DNA was detected by real-time PCR of non-invasive diagnostic samples (pharyngeal and nasal swabs) and in BAL immediately after exposure and for 7 days after exposure. This is a novel finding and it is key in that, although this was a high-dose aerosol exposure not likely to be replicated in nature, obtaining both positive cultures and PCR detection from such samples argues for including PCR for *Brucella* in the diagnostic algorithm when an apparent point-source outbreak of respiratory illness occurs and cannot otherwise be diagnosed.

*Brucella* was never isolated from or detected by PCR of blood, serum or urine. Failure to detect *Brucella* in blood components or urine correlates well with human clinical diagnostics experience. Culture and PCR showed a progression of the pathogen from lung to spleen and liver, and finally to the bone marrow over the 7 day course of the experiment (Tables 1 and 2). In human brucellosis, it is recognized that *Brucella* quickly disseminates to the lymphatics as well.

Notably, PCR was positive in the gonads by day 5 in all cases, in both sexes (though, by chance, the animals remaining in the experiment by day 5 included only one male animal). This correlates well with the propensity for *B. suis* to localize to the testes in humans. PCR was not well correlated with gross or histopathology findings in the mesenteric lymph nodes, but did accord well with tracheo-bronchial lymph node pathology and became positive in a progressively larger number of mandibular lymph nodes throughout the 7 day course of the study. There was also frequent detection by PCR in the large intestine and kidney later in the study, perhaps indicating wide-ranging tissue dissemination. It quickly became impossible to detect *Brucella* DNA in buccal swabs, indicating that it does not remain in the mouth. Surprisingly, PCR was also frequently positive in face, conjunctival and aural swabs throughout the 7 day course of the study; however, these would not be likely diagnostic samples in a human outbreak situation, and, at least in the case of face and aural swabs, this probably did not indicate the presence of viable organisms, but rather may indicate only the presence of pathogen DNA in the oily or waxy residues of the face or ear canal. These samples were not cultured.

PCR and culture results matched in all cases for lung, liver and spleen. *Brucella* was cultured from one BAL sample on day 7, and from two pharyngeal swab samples on each of days 5 and 7, while DNA was not detected by PCR. *Brucella* DNA was detected by PCR in bone marrow in one case on day 5, in the pharyngeal swab in one case on day 1 and in the nasal swab in one case on day 3 and two cases each on days 5 and 7, in which culture was not positive. All diagnosis mis-matches were in cases where relatively lower amounts of bacteria were recovered or the PCR threshold crossing value was relatively higher, indicating lower levels of *Brucella* or *Brucella* DNA at the lower limit of detection, wherein both methods become unreliable.

Overall, these results indicate that a battery of tests on a maximum number of sample types should be conducted to optimize the chances of detecting *Brucella*. However, when *Brucella* is not suspected in an outbreak situation, yet no other diagnostic methodologies yield a diagnosis, screening for *Brucella* by PCR may prove rewarding. Based on these results, neither culture nor PCR can be considered ideal for diagnosis of individual cases; however, the discrepancies in no way reduce the validity of including PCR in the diagnostic regimen for diagnosing an outbreak. It is never normal to either detect *Brucella* DNA or recover it by culture in a human being. Therefore, any positive result would be cause for further investigation. PCR is the ideal method of the two for screening because it is vastly faster and largely obviates biosafety problems that result from *Brucella* culture.

### Telemetry data

**Fig. 1.**
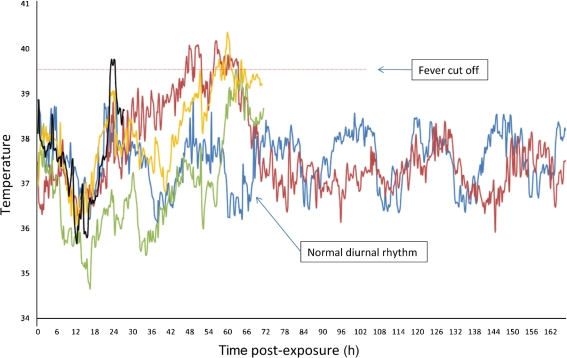
Temperature trends in four monkeys that showed fever spikes (black, red, yellow and green lines). Lines terminate on days on which animals were euthanized according to the protocol schedule.

The intervention parameter, which distinguished baseline temperatures from temperatures immediately prior to euthanasia, was not statistically significant for those subjects euthanized on day 3 (*P*=0.9861) but was significant for those subjects euthanized on days 5 (*P*=0.0270) and 7 (*P*=0.0037). Fever spikes were detected in single monkeys on days 1 and 2 and in three monkeys on day 3 (Fig. 1). Fever spikes were not noted on any other days. Fever was never detected in control monkeys. Although the rhesus model does not allow assessment of fever-related manifestations of brucellosis that occur in humans (e.g. night sweats), waxing and waning fever is consistent with human brucellosis and indicates that the rhesus macaque is a good model for human brucellosis.

No change in activity levels was detected. No significant CBC or blood chemistry aberrations were detected. This is also consistent with human brucellosis because clinical pathology indicators are indistinct in human brucellosis (Demirtürk *et al.*, 2008).

### Gross pathology

In terms of gross pathology, from day 1 post-exposure through day 7, there were varying degrees of enlargement of the mesenteric, tracheobronchiolar and mandibular lymph nodes. In all cases there was mild oedema. There were no significant gross findings in the remaining organs.

On day 1 post-exposure, the most significant gross findings were enlarged and slightly oedematous mesenteric lymph nodes (two to three times normal). By day 3, mesenteric lymph nodes were enlarged by up to five times normal and remained mildly oedematous. At that time point, the tracheobronchial lymph nodes were enlarged up to three times normal and the mandibular lymph nodes were also slightly enlarged in two of the three monkeys sampled on that day.

On day 5, the mesenteric lymph nodes were still oedematous and enlarged, but only up to three times normal. The tracheobronchial lymph nodes were enlarged three to four times normal and congested. The mandibular lymph nodes were slightly enlarged. By day 7, the mesenteric lymph nodes were enlarged two to three times normal and oedematous. The tracheobronchial lymph nodes were enlarged up to four times normal and oedematous. The mandibular lymph nodes were still slightly enlarged.

Enlarged lymph nodes were occasionally noted in the controls; however, oedema was not noted. Lung congestion was noted in two of three exposed monkeys euthanized on days 1 and 3 after exposure, and in one of three monkeys euthanized on days 5 and 7 after exposure.

The mesenteric lymph nodes were the first to show any significant macroscopic changes. By day 3, they were their largest over the span of 7 days. The tracheobronchial lymph nodes did not show any significant changes until day 3 and then reached the maximum noted size by day 7. These findings were non-specific, and are consistent with varying degrees of antigenic stimulation that corresponds to the lymphoid hyperplasia noted histologically (see below). There was no corresponding pattern of lymph node enlargement in the controls as was noted in the exposed animals. There was more oedema of the lymph nodes in the exposed animals than in the controls. Oedema is often an early (acute) manifestation of inflammation and may occur prior to any other grossly detectable indication of inflammation (Kumar *et al.*, 2005). The lung congestion noted was likely a terminal event related to the method of euthanasia. All gross and histological findings were non-specific and would not be attributable to any specific agent this early in the course of the disease.

### Histology

In terms of histological findings, the lymphoid hyperplasia and lymph node oedema corresponded with the gross findings of lymph node enlargement and oedema. There were no other significant histological findings specifically attributable to exposure to aerosolized *B. suis.* On day 3, there was multifocal bronchiolar epithelial degeneration and necrosis, with mild lymphohistiocytic and neutrophilic bronchiolitis and peribronchiolitis with alveolar oedema. The inflammation worsened by day 5 and the oedema was more pronounced. By day 7, the inflammation was similar to that observed on day 5 but the perivascular oedema was slightly more pronounced. By day 7, two out of three rhesus macaques showed mild lymphohistiocytic hepatitis.

All tissues were stained with haematoxylin and eosin only and there was no attempt to visualize *Brucella* organisms in tissues. This corresponds with the approach that would be expected to be taken in diagnosing a biopsy specimen from an unknown case of acute febrile illness. There is virtually no information in the literature concerning histological findings in the lymph nodes of human patients. Occasionally, brucellosis has manifested as hepatitis, so there is a slight possibility that a liver biopsy might be a primary diagnostic sample for brucellosis, but there is almost no chance that a biopsy would be taken early in the course of infection because hepatic enlargement or changes notable on ultrasound are not reported to occur early in the course of exposure (Akritidis *et al.*, 2007). Based on the limited information available, our data are consistent with reports of liver histology in human brucellosis, i.e. that parenchymal necrosis and lymphocytic infiltration are common. Histological findings in pulmonary brucellosis cases vary, and the vast majority of information available in the literature is associated with chronic cases, but our data are consistent with the limited amount available, i.e. that inflammation is often lymphohistiocytic and/or neutrophilic (Theegarten *et al.*, 2008). We did not observe progression to granulomatous inflammation, presumably because of the short duration of the study. However, in fact, the histology findings in our study are meaningful for their indistinctiveness; that is, in contrast to later stage *B. suis* infection, in this model of acute *B. suis* infection, no major histological abnormalities were observed. Therefore, alternative diagnostic methods such as PCR would be expected to be preferable due to their higher speed and throughput. In other words, when faced with an unknown, the diagnostician may use PCR as a screening method.

### Conclusions

Taken together, these data indicate that the rhesus macaque is a good model of human brucellosis. If this is the case, then an intentional human exposure would not be associated with any distinctive clinical, haematological or pathological signs. There would be little basis to suspect any aetiology, let alone brucellosis. For this reason, a rapid, reliable screening test is essential. Our data indicate that an intentional human exposure by aerosol can be detected by PCR of non-invasive samples, i.e. nasal and/or pharyngeal swabs. We detected *Brucella* DNA in nasal swab samples in all three monkeys sampled at each time point (days 1, 3, 5 and 7 after exposure – with progressively declining apparent concentration based on the mean PCR threshold crossing points) and in pharyngeal swabs in two cases on day 1, three cases on day 3 and one case on day 5. DNA concentrations were not standardized; all methods were conducted in a manner similar to that which would be expected in a clinical diagnostic laboratory, i.e. qualitative.

In an intentional exposure scenario, it is reasonable to expect a wide range of exposure doses, such that a subset of individuals would inhale a high dose; the far end of this spectrum was modelled in this study. Heavily exposed humans would probably experience upper respiratory illness and potentially enlarged regional lymph nodes. Physicians commonly attempt diagnosis of such cases with nasal and/or pharyngeal swabs, which are submitted for culture. Culture could also be rewarding in high-dose exposure cases, but PCR would provide a diagnosis within hours whereas culture would require days. The PCR assay used here is highly specific for the *Brucella* genus and *Brucella* is never normal flora for humans, so a PCR-positive sample would provide a high degree of confidence that a diagnosis had been made. The primary importance of these data is to show that adding PCR assays for selected agents can result in a diagnosis in an otherwise confusing outbreak situation, in a timely enough fashion that successful treatment and attribution may be possible. It would be reasonable in this scenario to empirically treat symptomatic individuals that may also have been exposed, but for which no definitive diagnosis could be obtained. Diagnosis of individuals exposed to a lower dose may still require invasive methods such as lymph node or bone marrow biopsy. In this study, neither culturable organism nor bacterial DNA could be found in blood, urine or any other non-invasive sample except certain swabs.

In contrast to the study conducted by Mense *et al.* (2004), this study shows for the first time that high-dose *Brucella* exposure of rhesus macaques can result in rapid illness and early dissemination to the entire haematopoietic system, liver and gonads. The diagnostic methods are expected to be applicable to other pathogenic *Brucella* species. *B. melitensis*, *Brucella abortus* and *B. suis* cause the vast majority of human disease. *Brucella canis* has been associated with human disease only in immunosuppressed patients, and marine mammal *Brucella* species have been associated with isolated cases of human disease but speciation continues to be debated (Aleixo *et al.*, 1999; Maquart *et al.*, 2008; Whatmore *et al.*, 2008). *B. abortus* was once common in the United States and caused hundreds of cases of human brucellosis annually, before being largely eradicated toward the end of the last century. *B. melitensis* is generally regarded as the most important species because it is associated with most severe human cases; however, this is due in part simply to its greater prevalence in key animal reservoirs. *B. suis* also causes severe disease in humans, but *B. melitensis* causes more obvious, ‘classical’ undulant fever while *B. suis*-associated disease is often associated with abscess formation (Young, 1983). Laboratory-acquired infection is a common cause of brucellosis and historical evidence of laboratory exposure and infection indicates that the infectious dose of *Brucella* is extremely low. *B. suis* was weaponized by the US, former Soviet Union and China (Hoover & Friedlander, 1997) and this was an important factor in our decision to model *B. suis* infection as opposed to any other species of *Brucella*. A further reason for choosing to perform these studies with *B. suis* as opposed to *B. melitensis* or *B. abortus* is that the main surface antigen of *B. abortus* (so-called ‘A’ antigen) and that of *B. melitensis* (so-called ‘M’ antigen) are both present in *B. suis*. Therefore, *B. suis*-infected tissues archived during the course of this study can serve as a test set for future diagnostic assays specifically designed to detect either antigen.

## Figures and Tables

**Table 1.** Log_10_ c.f.u. of bacteria (g tissue)^−1^ or (ml fluid)^−1^ detected from *B. suis*-infected rhesus macaques Numbers in parentheses denote the number of animals out of a total of three per time point in which *B. suis* was detected, if less than three. Blood and serum were also cultured but no organism was detected from these fluids. Data are reported as mean±standard error of the mean.

**Tissue/sample type**	**Day**			
	**1**	**3**	**5**	**7**
Lung	3.16±0.86	5.19±0.79	4.66±0.57	5.31±0.49
Liver			0.75±0.16 (2)	1.60±0.15
Spleen			1.85±0.59 (2)	3.32±0.41
Bone marrow				0.98±0.26
Pharyngeal swab	3.91±0.43	4.14±0.35	3.66±0.17	2.84±0.15 (2)
Nasal swab	2.27±0.16	3.93±0.43 (2)	1.85 (1)	5.04 (1)
Broncho-alveolar lavage	5.70±1.84	2.50±0.26	2.25±0.40	1.31±0.01

**Table 2.** PCR results from tissues or other samples from *B. suis*-infected rhesus macaques Numbers denote the mean threshold crossing point of a triplicate for a given animal (three per time point, except in the case of reproductive organs, where the number per time point is indicated in parentheses). Brain, blood, serum, urine and small intestine were never positive.

**Tissue/sample type**	**Day**			
	**1**	**3**	**5**	**7**
Pharyngeal swab	37.2, 38.0	33.8, 36.7, 36.8	38.0	
Nasal swab	25.8, 29.2, 29.3	30.0, 34.6, 36.2	32.9, 34.4, 36.0	34.8, 36.3, 37.6
Face swab	27.7, 27.8, 28.7	32.1, 30.3	30.5, 34.0, 34.4	32.6, 33.7, 34.2
Conjunctival swab	30.0, 30.7, 32.5	31.0, 33.6, 36.4	31.7	33.7, 34.3, 36.1
Buccal mucosal swab	35.5	36.7		
Aural swab	29.1, 29.5, 29.8	30.4, 30.9, 32.7	29.6, 31.7, 33.9	33.0, 33.2, 34.4
Heart	37.2			37.5
Kidney			34.2, 36.9, 37.9	38.0
Large intestine	34.1		31.1, 33.5, 34.5	31.9, 34.6
Testes	(1)	(1)	35.6 (1)	
Epididymis	(1)	(1)	33.0 (1)	
Prostate	(1)	(1)	34.3 (1)	
Ovary	(2)	(2)	36.2, 36.4 (2)	33.7, 35.5, 35.7 (3)
Uterine tube	(2)	(2)	(2)	(3)
Uterus	(2)	(2)	(2)	(3)
Mesenteric lymph node				36.4, 36.8
Tracheobronchial lymph node	29.2, 32.2, 32.8	24.6, 29.2, 29.7	26.3, 27.0, 27.1	26.8, 29.7, 34.1
Mandibular lymph node		29.6	27.5, 34.1	30.6, 31.8, 33.4
Lung	23.5, 26.7, 35.7	22.3, 24.6, 25.7	23.3, 24.2, 24.9	24.3, 24.7, 27.5
Liver			34.6, 35.5	32.3, 33.8, 34.1
Spleen			32.3, 38.4	30.8, 34.3, 35.3
Bone marrow			35.7	34.2, 35.9, 36.1
Broncho-alveolar lavage	29.0, 31.0, 31.3	32.7, 33.6, 35.1	34.0, 35.6, 36.2	34.9, 36.4

## Abbreviations

- BAL, broncho-alveolar lavage
- CBC, complete blood count
